# Antiparasitic Effects of Asteraceae Species Extracts on *Echinococcus granulosus* s.s

**DOI:** 10.1155/2022/6371849

**Published:** 2022-09-19

**Authors:** C. M. Albani, J. Borgo, J. Fabbri, P. Pensel, A. Paladini, M. F. Beer, L. Laurella, O. Elso, N. Farias, N. Elissondo, G. Gambino, V. Sülsen, M. C. Elissondo

**Affiliations:** ^1^Instituto de Investigaciones en Producción Sanidad y Ambiente (IIPROSAM CONICET-UNMdP), Facultad de Ciencias Exactas y Naturales–UNMdP, Centro Científico Tecnológico Mar Del Plata–CONICET, Centro de Asociación Simple CIC PBA, Mar Del Plata, Argentina; ^2^Laboratorio de Zoonosis Parasitarias, Facultad de Ciencias Exactas y Naturales (FCEyN), Universidad Nacional de Mar Del Plata (UNMdP), Mar Del Plata, Buenos Aires, Argentina; ^3^Universidad de Buenos Aires, CONICET, Instituto de Química y Metabolismo Del Fármaco (IQUIMEFA), Buenos Aires, Argentina; ^4^Universidad de Buenos Aires, Facultad de Farmacia y Bioquímica, Departamento de Farmacología, Cátedra de Farmacognosia, Buenos Aires, Argentina; ^5^Cátedra de Parasitología Comparada, Facultad de Ciencias Veterinarias (UNLP), Mar Del Plata, Buenos Aires, Argentina; ^6^Laboratorio de Invertebrados, Instituto de Investigaciones Marinas y Costeras (IIMYC) (UNMDP-CONICET), Argentina; ^7^Laboratorio de Análisis Clínicos, Santisteban 7000, Tandil, Buenos Aires, Argentina

## Abstract

Cystic echinococcosis is a zoonotic disease caused by the parasite *Echinococcus granulosus* sensu lato (s.l.), which is worldwide distributed and causes long-lasting infections in animals and humans. The existing treatment is limited to the use of benzimidazoles, mainly albendazole (ABZ). However, it has unwanted side effects and its efficacy is about 50%. The Asteraceae family includes plants that have therapeutic applications (medicinal species) and has an important role in new drug development. The species belonging to a different genus of this family show a wide range of anti-inflammatory, antimicrobial, antioxidant, hepatoprotective, and antiparasitic activities, among others. The aim of the present study was to evaluate the *in vitro* efficacy of extracts of four Asteraceae species against protoscoleces of *E. granulosus* sensu stricto (s.s.). On the other hand, the *Stevia aristata* extract was assessed on the murine cyst of *E. granulosus* (s.s.) and the efficacy of *S. aristata* extract was investigated in a murine model of CE. *Stevia satureiifolia*, *S. aristata*, *Grindelia pulchella,* and *G. chiloensis* extracts at 100 *μ*g/mL caused a decrease in protoscoleces viability; however, *S. aristata* extract produced the greatest *in vitro* protoscolicidal effect. After 20 days of treatment with the highest concentration (100 *μ*g/mL) of *S. aristata* extract, protoscoleces viability decreased to 0%. The tegumental changes observed by scanning electron microscopy were consistent with the reduction in vitality. The collapse of the germinal layer was registered in 60 ± 5.8% and 83.3 ± 12.0% of cysts treated during 4 days with 50 and 100 *μ*g/ml, respectively. The half maximal effective concentration (EC50) value of the *S. aristata* extract against *E. granulosus* (s.s.) cysts was 47.86 *μ*g/mL (96 h). The dosage of infected animals with the 50 mg kg^−1^ dose of *S. aristata* extract resulted in a significant reduction in cyst weight in comparison with the control group. In conclusion, *S. aristata* extract was demonstrated to exert a marked effect, both in vitro and in the murine model.

## 1. Introduction

*Echinococcus granulosus* sensu lato (s.l.) (henceforth *E. granulosus*) is the causal agent of cystic echinococcosis (CE), a zoonotic disease worldwide distributed. This tapeworm is naturally passed between canid definitive hosts (like dogs) that harbor the adult stage in the intestine, and mostly ungulate intermediate hosts with larval cysts (metacestodes) developing in their internal organs. Definitive hosts are infected by ingestion of parasite larvae, mostly by feeding on the infected offal of livestock [1]. Humans acquire the infection when ingesting *E. granulosus* eggs after hand-to-mouth contact with contaminated matrices, such as egg-contaminated dog fur or soil, and by consumption of contaminated water or food [2]. The burden of disease on affected people is often life-long and the costs of medical attention in endemic countries are significant [3].

Currently, available treatment options for CE based on the cyst stage classification proposed by WHO-IWGE (World Health Organization Informal Working Group on Echinococcosis) include (i) surgery, (ii) percutaneous treatment including the puncture, aspiration, injection, reaspiration (PAIR) technique, (iii) antiparasitic treatment with albendazole (ABZ), and (iv) observation with no intervention for nonactive cysts [4].

Systemic anti-infective treatment is based on the continuous administration of albendazole (ABZ) or mebendazole, being the only anti-infective drug clinically effective in interrupting *Echinococcus spp*. larval growth [4]. Due to ABZ increased bioavailability and easier administration to patients, it is the preferred anti-infective treatment for echinococcosis, at an average dosage of 15 mg/kg/day [5]. However, it has been shown that BMZ treatment was associated with a high rate of relapses depending on the cyst type, age of the patient, and the organ affected, exhibiting a parasitostatic effect [6]. On the other hand, hepatotoxicity, alopecia, gastrointestinal disturbances, thrombocytopenia, and severe leukopenia have been reported, and these cases require stopping the prescribed treatment [7]. New alternative treatments are urgently required based on pharmacological evidence and the relatively low and slow efficacy of ABZ [8].

In recent years, a growing number of plant-derived products have been tested against CE in an attempt to find alternative natural compounds for effective treatment [4]. Nonetheless, the vast majority of these molecules have been tested *in vitro* against *E. granulosus* protoscoleces and only a few have been evaluated for their in vivo activity in the murine model [9].

The Asteraceae family is one of the most diverse flowering plant families, with members having therapeutic applications and playing an important role in the development of new medicines. They exhibit a wide range of biological activities, including anti-inflammatory, antimicrobial, antioxidant, hepatoprotective, and antiparasitic properties. The pharmacological effects of these plants can be attributed to their presence of different phytochemical compounds, including flavonoids, polyphenols, phenolic acids, acetylenes, and terpenoids, among others [10].

In particular, the genus Stevia represents one of the most diverse and characteristic of the family Asteraceae. Its range extends from the southern United States to the Andean region of South America, as well as northern Chile and northern Patagonia in Argentina [11], and it grows in a variety of environments [12]. Extracts and isolated compounds from the *Stevia* species have been shown to have a variety of biological activities. The majority of them have antioxidant, antiparasitic, antiviral, anti-inflammatory, and antiproliferative properties [13].

Based on the promising findings recently reported by Albani et al. [14] where a *Stevia multiaristata* extract was used against *E. granulosus* (s.s.), we proposed to carry out a screening of other Asteraceae species.

For this purpose, the aim of the present study was to evaluate the *in vitro* efficacy of *Stevia satureiifolia*, S. aristata, *Grindelia pulchella,* and *G. chiloensis* extracts against protoscoleces of *E. granulosus* (s.s.). On the other hand, the *S. aristata* extract was assessed on the murine cyst of *E. granulosus* (s.s.) and the *in vivo* effect of this extract was investigated in a murine model of CE.

## 2. Material and Methods

### 2.1. Plant Material

Specimens were collected from different locations throughout Argentina. *Grindelia chiloensis* (Cornel.) Cabrera (Asteraceae) aerial parts were collected in Neuquén province in January 2019. *Stevia satureiifolia* var. *satureiifolia* (Lam.) Sch. Bip. ex Klotzsch (Asteraceae) was collected in Buenos Aires province in February 2012. *Stevia aristata* D. Don ex Hook. and Arn. (Asteraceae) and *Grindelia pulchella* Dunal (Asteraceae) were collected in Entre Ríos province in December 2012 and March 2019, respectively.

Voucher specimens (Number: BAF 16000, BAF 744, BAF 797, and BAF 14869, respectively) are available at the Museum of Pharmacobotany, Faculty of Pharmacy and Biochemistry, University of Buenos Aires.

### 2.2. Extraction

Crude extracts of *G. chiloensis, S. aristata, S. satureiifolia*, and *G*. *pulchella* were prepared by maceration of the dried aerial parts of the plants with dichloromethane (10% *w/v*) at room temperature. The extracts were filtered using filter paper and dried in a rotary evaporator under a vacuum.

#### 2.2.1. Drugs Preparation

For *in vitro* studies, *S. satureiifolia, S. aristata, G. pulchella,* and *G. chiloensis* extracts were prepared in dimethyl sulphoxide (DMSO) at a concentration of 40 mg/mL.

For the clinical efficacy study, ABZ (Pharmaceutical grade, Parafarm, Argentina) suspension (5.25 mg/mL) was dissolved in distilled and deionized water as reported by [15]. *S. aristata* extract (6.66 mg/mL) was prepared in olive oil using DMSO (0.5%*v/v*).

#### 2.2.2. Parasite Material

Protoscoleces of *E. granulosus* were collected aseptically from the hydatid cysts of the liver and lungs of the naturally infected cattle. Protoscoleces were isolated from fertile cysts following Elissondo et al. [16]. Protoscoleces viability was assessed by employing the vital dye methylene blue [17]. Genotyping of the parasitic material was carried out as previously described by Cucher et al. [18] Based on sequencing analysis, the G1 genotype was identified.

Four CF-1 female mice were infected by an intraperitoneal injection of 1,500 *E. granulosus* (s.s.) protoscoleces in 0.5 mL of medium 199 (Lab. Microvet SA, Argentina) to obtain murine cysts. After 6 months of inoculation, mice were anesthetized with ketamine (100 mg/kg) and xylazine (10 mg/kg), euthanized, and necropsied. After that, the cysts were carefully recovered from the peritoneal cavity [19].

### 2.3. *In Vitro* Treatment of Protoscoleces and Cysts

Protoscoleces (2000 per Leighton tube) were incubated in 6 mL of culture medium 199 at 37°C [16] and different extracts were added at final concentrations of 100 *μ*g/mL. After the screening of the different extracts, and based on the obtained results, protoscoleces were incubated with 100, 50, 10, and 5 *μ*g/mL of *S. aristata* extract.

In all cases, tubes were followed every day using an inverted microscope and the occurrence of morphological changes were determined. Viability was assessed periodically using the vital dye methylene blue.

Cysts (10 per group) were placed in Leighton tubes with 6 mL of medium 199 and different concentrations of the *S. aristata* extract (100, 50, 10, and 5 *μ*g/mL. Tubes were maintained at 37°C and macroscopic and microscopic changes such as loss of turgidity and the collapse of the germinal layer were registered daily. In all cases tubes containing 3 *μ*L/mL of DMSO were used as control. Experiments were performed in triplicate and repeated three times.

#### 2.3.1. Treatment Efficacy on Mice

Thirty mice were infected by an intraperitoneal injection of *E. granulosus* protoscolesces as was previously described in the section “Parasite material.” After 6 months of postinfection, mice were divided among 3 experimental groups (10 animals/group) and treated as follows: (1) Control group; (2) ABZ group and animals treated with ABZ suspension (25 mg/kg) for 30 days; (3) *S. aristata* group, animals treated with the extract (50 mg/kg) for 23 days. Treatments were performed by intragastric administration, every 24 h.

At the end of the experiment, animals were anesthetized with a mixture of ketamine (100 mg/kg) and xylazine (10 mg/kg), and blood samples were collected by cardiac puncture for biochemical studies. Then mice were euthanized and cysts from the peritoneal cavity were carefully removed. The weight of the cysts recovered from each individual animal was recorded. Samples of cysts from each group were fixed and processed for SEM.

### 2.4. Biochemical Methods

Blood was centrifuged (2000 × g for 15 minutes) and plasma was separated and stored at −20°C until further analysis. Commercial kits were used to quantify the enzymes alkaline phosphatase (ALP), gamma-glutamyl transpeptidase (GGT), and glutamate-pyruvate transaminase (GPT) (Wiener Lab, Rosario, Argentina ).

The estimation of the enzymes GGT, GPT, and ALP was done by the modified Szasz method, the UV method, and the method described by Bessey et al. [20], respectively [21–23].

#### 2.4.1. Electron Microscopy

Samples of protoscoleces collected from the different *in vitro* treatments and cysts retrieved from the different *in vitro* or *in vivo* studies were processed for SEM following the protocol previously described by Elissondo et al. [19].

### 2.5. Statistical Analysis

A generalized linear model (GLM) with a binomial distribution of the error was fitted for the *in vitro* treatment of protoscoleces with the different extracts. The proportion of viability was the response variable and treatments and time in days were the explanatory variables. The “ANOVA” command from the “car” package was used to assess whether time-treatment interactions were required to be included in the model [24]. Pairwise contrasts of the interaction mean using the “emmeans” package was used to assess the differences among the *S. aristata* extract concentrations and control [25].

For the *in vitro* incubation of cysts with *S. aristata* extract, differences between treatments and exposure times were tested by fitting analysis of the variance model with the percentage of cysts with germinal layer collapse as a response variable and extract concentration and exposure time as explanatory variables. Then a Tukey HSD test was applied for pairwise contrasts. Moreover, the half maximal effective concentration (EC50) of the extract was calculated using the “ec50estimator” package [26].

Differences in the weight of the cysts and the plasma levels of ALP, GGT, and GPT enzymes between *in vivo* treated groups were assessed by Mann–Whitney and Kruskal–Wallis tests, respectively. The weights of the cysts for each treatment are reported as the median and interquartile range (IQR).

All statistical analyses were conducted within the *R* environment [27] considering ^*∗*^*p* values less than 0.05 as statistically significant.

### 2.6. Ethic Statement and Experimental Animals

All procedures and management protocols carried out involving animals were approved by the Institutional Animal Care and Use Committee (RD N 211/2018) of the Faculty of Exact and Natural Sciences, National University of Mar del Plata, Argentina. All the proceedings were performed following the revised form of the Guide for the Care and Use of Laboratory Animals (National Research Council US, 2011). Female CF-1 mice (*n* = 34) and body weight (25 *g* ± 5) were used. The animals were housed in a temperature-controlled (22 ± 1°C) and light-cycled (12 h light/dark cycle) room. Food and water were given ad libitum.

## 3. Results

### 3.1. *In Vitro* Incubation of Protoscoleces with the Different Extracts

**Figure 1:**
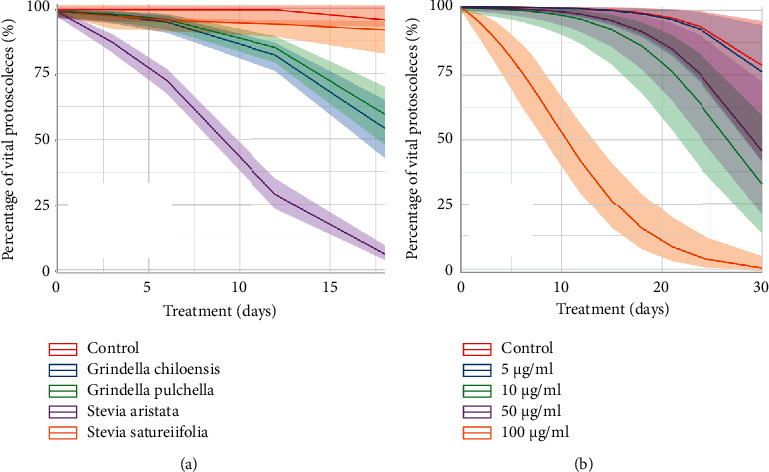
Estimates of *Echinococcus granulosus* (s.s.) protoscoleces survival after *in vitro* exposure with (a) different extracts of Asteraceae species at 100 *μ*g/mL, (b) different concentrations of *S. aristata* extract. The lines and ribbons indicate the predicted fits and 95% confidence intervals from a generalized linear mixed-effects model.

Figure 1(a) shows the survival of protoscoleces after exposure to different extracts of Asteraceae species at 100 *μ*g/mL. Although all studied extracts caused a decrease in viability, *S. aristata* produced the greatest protoscolicidal effect.

Figure 1(b) shows the survival of protoscoleces after exposure to different concentrations of *S. aristata* extract. Control protoscoleces incubated in the absence of a drug remained viable (78% (42%–95%)) after 30 days of incubation. Protoscoleces cultured with 100 *μ*g/mL of *S. aristata* extract were killed considerably faster than protoscoleces cultured with 5, 10 or 50 *μ*g/mL of *S. aristata* extract. After 10 days of exposure with 100 *μ*g/mL of *S. aristata* extract, the viability was approximately 46% and reduced to 0% after 20 days of incubation. *S. aristata* extract at concentrations of 5, 10 and 50 *μ*g/mL provoked a later protoscolicidal effect, causing a reduction in protoscoleces viability of 76% (42%–93%), 33% (15%–59%), and 46% (22%–72%), respectively, after 30 days p.i (Figure 1(b)).

**Figure 2:**
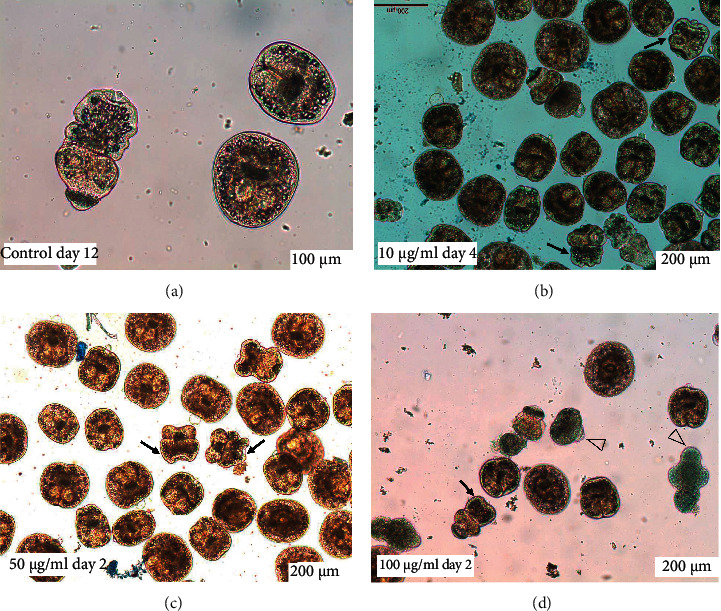
Images at the light microscope of *E. granulosus* (s.s.) protoscoleces treated *in vitro* with 50 *μ*g/mL and 100 *μ*g/mL of *S. aristata* extract. Stained protoscoleces (blue) are not viable. Arrows point out the contracted soma and arrowheads show the rostellar disorganization.

**Figure 3:**
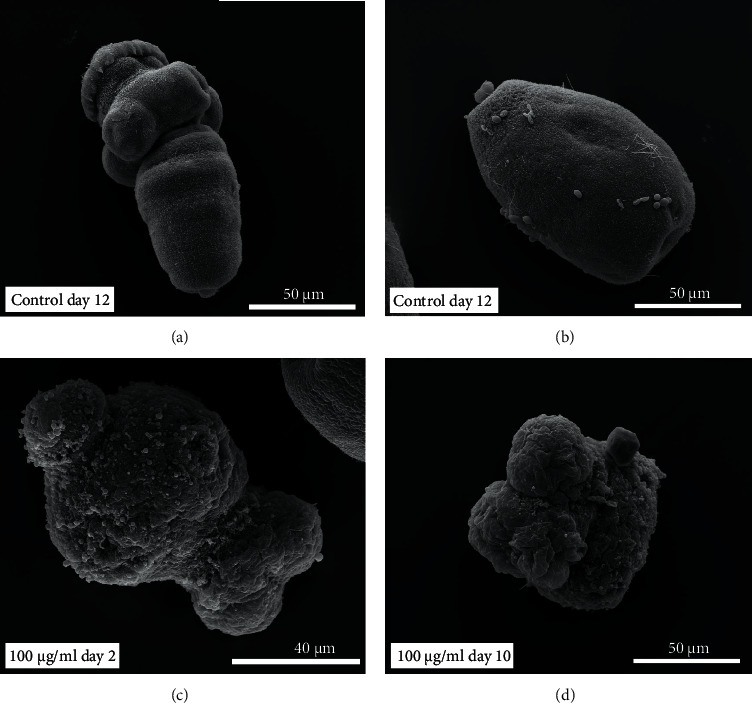
Images at scanning electron microscope of *E. granulosus* (s.s.) protoscoleces treated in vitro with 100 *μ*g/mL of *S. aristata* extract. Note untreated protoscoleces exhibiting an intact morphology both evaginated (a) or invaginated (b)-(c) Protoscolex incubated with 100 *μ*g/mL of *S. aristata* extract for 2 days showing contraction of the soma region, extensive damage in the tegument, and loss of microtriches and hooks. (d) Protoscolex treated with 100 *μ*g/mL of *S. aristata* extract for 10 days showed total loss of morphology.

Throughout the incubation period, control protoscoleces showed no structural or ultrastructural changes, displaying an intact morphology whether evaginated or invaginated (Figures 2(a) and 3(a) and 3(b)). In contrast, morphological and ultrastructural damage were detected in protoscoleces treated with *S. aristata* extract. After 2 days p.i., soma contraction was observed in protoscoleces treated with 50 *μ*g/mL (Figure 2(c)). The same alteration was observed in protoscoleces treated for 4 days with 10 *μ*g/mL (Figure 2(b)). The treatment with 100 *μ*g/ml of *S. aristata* extract for 2 days caused contraction of the soma region, extensive damage in the tegument, and loss of microtriches and hooks (Figures 2(d) and 3(c)). Total loss of morphology was observed after 10 days of treatment with the same concentration (Figure 3(d)).

*In vitro* treatment of cysts with *S. aristata* extract.

**Figure 4:**
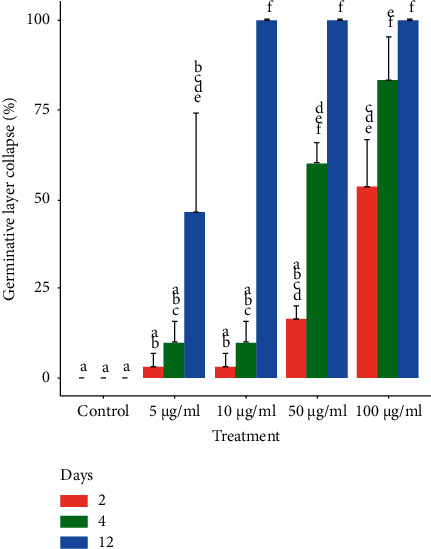
Effect of different concentrations of *S. aristata* extracts on *E. granulosus* (s.s.) cysts after 2, 4, and 12 days of in vitro exposure. The collapse of the germinal layer was the criteria used to evaluate the cysticidal effect. Different letters above the bars indicate statistically significant differences at ^*∗*^*P* < 0.05. Each bar depicts the mean percentage ± SD (standard deviation).

**Figure 5:**
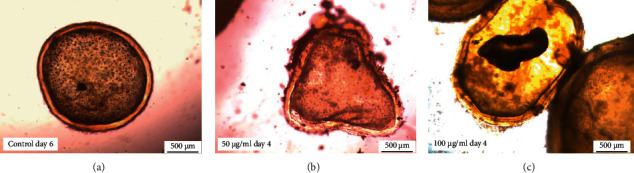
Images at the light microscope of *E. granulosus* (s.s.) cysts treated *in vitro* with 50 *μ*g/mL and 100 *μ*g/mL of *S. aristata* extract. Observe (a) the typical morphology of the untreated cyst, (b) the loss of turgidity, and (c) the collapse of the germinal layer.

Throughout the *in vitro* experiment, control cysts appeared macroscopically turgid, with no observable collapse of the germinal layer (Figures 4 and 5(a)). In contrast, the collapse of the germinal layer was observed in 60 ± 5.8% and 83.3 ± 12.0% of cysts treated for 4 days with 50 and 100 *μ*g/ml, respectively (Figures 4 and 5(b) and 5(c). The treatment of cysts with 5 and 10 *μ*g/mL caused a delayed effect (Figure 4).

The *E. granulosus* (s.s.) cysts incubated with *S. aristata* extract showed an EC50 value of 47.86 *μ*g/mL (96 h).

#### 3.1.1. Treatment Efficacy on Mice

Throughout the experiment, the animal's behavior and appearance were normal. Furthermore, no statistical differences in ALP, GGT, and GPT activities were found between control and treated mice (*P* < 0.05).

All the infected animals involved in the clinical efficacy study developed hydatid cysts.  Table 1 summarizes the cyst weights (median and IQR) recorded after treatments of the different experimental groups involved in the clinical efficacy study. Although the median weight of cysts recovered from ABZ-treated mice was lower than that observed in the control group, no significant differences were found (*P* < 0.05). The weight of the cysts significantly decreased after *S. aristata* treatment than that in the control group (*P* < 0.05).

**Figure 6:**
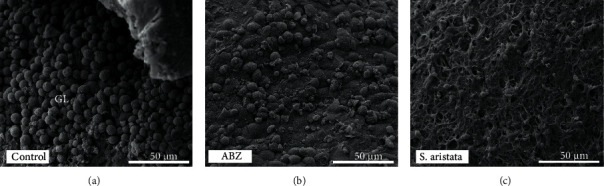
Images at scanning electron microscope of *E. granulosus* (s.s.) cysts recovered after clinical efficacy study. CF-1 female mice were treated with ABZ suspension (25 mg/kg) or *S. aristata* extract (50 mg/kg) every 24 h. Observe (a) cyst recovered from the control group where an intact germinal layer is present; (b) cyst recovered from mice treated with ABZ suspension showing loss of cells and presence of cellular debris; (c) cyst recovered from mice treated with *S. aristata* extract where a totally impaired germinal layer is shown.

Figure 6 shows the ultrastructural analysis of the germinal layer of metacestodes retrieved from the control and treated groups. Cysts obtained from control mice showed the characteristic multicellular structure of the germinal layer (Figure 6(a)). Metacestodes recovered from medicated mice showed damage in the germinal layer, in relation to the control group. However, the damage extension appears to be greater after *S. aristata* extract treatment (Figure 6(c)) than that after ABZ treatment (Figure 6(b)).

## 4. Discussion

Cystic echinococcosis (CE) is a neglected disease that causes severe health and economic problems [28]. Currently, the antiparasitic treatment of CE involves the use of benzimidazoles (BZM), with albendazole (ABZ) being the most commonly used. However, this treatment option is not curative and it often leads to side effects [7]. Therefore, new pharmacotherapeutic options are needed in order to optimize the treatment of CE.

In the search for new agents for the treatment of CE, the activity of the compounds can be assayed *in vitro* and *in vivo* [4]. For the first *in vitro* screening of novel drugs, the targets include protoscoleces and metacestodes. In the next step, the active compounds could be tested in vivo. The murine model of cystic echinococcosis gives a more realistic approach to the study of antiparasitic drugs compared with *in vitro* experiments [8]. Like all experimental animal models, the murine model of cystic echinococcosis has advantages and disadvantages. The main strengths of the model are the small size of mice, the low cost of maintenance with respect to other mammals, and the shorter cyst development times. The major shortcoming is that the mouse is not a natural host for *E. granulosus* (s.s.). Another drawback is the location of the infection. The cysts develop within the peritoneal cavity and this no resembles the infection in the natural hosts. In spite of all these inconveniences, the experimental infection of mice with protoscoleces constitutes a widely used in vivo model for the assessment of the efficacy of different compounds against cystic echinococcosis [4].

Since ancient times, natural products, such as plant extracts, have been applied for medical treatments and are the basis of modern medicine due to having high availability, high efficacy, and low side effects [29]. This study aimed to evaluate the *in vitro* efficacy of different Asteraceae plant extracts against *E. granulosus* (s.s.) and to investigate the *in vivo* effect of *S. aristata* extract in a murine model of CE.

The Asteraceae family is widely distributed throughout the world and in a variety of habitats ranging from forests, high-altitude grasslands, and even urban green spaces [30]. Several members within the Asteraceae family have shown pharmacological activities, such as anti-inflammatory, antimicrobial, antioxidant, hepatoprotective, and antiparasitic activities, which have been attributed to their phytochemical constituents, such as essential oils, lignans, saponins, polyphenolic compounds, phenolic acids, sterols, polysaccharides, and terpenoids [31].

Within the Stevia genus, *Stevia rebaudiana* (Bertoni) Bertoni (Asteraceae), is worldwide known for its natural sweetener content. Preclinical and clinical investigations of *S. rebaudiana* have shown that it possesses a number of biological properties, including antidiabetic, anticariogenic, antioxidant, antihypertensive, antimicrobial, anti-inflammatory, and antitumor activities [32]. Moreover, the efficacy of *S. rebaudiana* against gastrointestinal parasites in a human was evaluated [33].

The present results demonstrated that *in vitro* treatment of protoscoleces with extracts of different members of the Asteraceae family *S. satureiifolia, S. aristata, G. pulchella,* and *G. chiloensis* caused a reduction in protoscoleces viability.

More specifically, the *in vitro* treatment of protoscoleces and murine cysts with the *S. aristata* extract induced a number of significant alterations that impaired parasite viability and led to its death. *S. aristata* extract caused a marked reduction in protoscoleces viability which was consistent with other distortions found as shrinkage of the soma region, extensive damage in the tegument, and loss and microtriches. In addition, *S. aristata* extract caused a rapid collapse of the germinal layer of the cysts. These results agree with our previous studies using *S. multiaristata* extract [14]. However, *S. aristata* extract caused a lower EC50 value (47.86 *μ*g/mL) at 96 h in comparison with *S. multiaristata* (69 *μ*g/mL).

Additionally, the ultrastructural modifications matched those shown in *E. granulosus* (s.s.) protoscoleces and cysts that had been treated *in vitro* with other drugs and natural products such as thymol, carvacrol, cinnamaldehyde, oregano, thyme, and cinnamon essential oils [15, 34–56].

A large number of plant extracts were tested in vitro on *E. granulosus* protoscoleces [9, 37–40]; however, only a few of them were investigated for preventive or therapeutic activities in the search for new alternative treatments for CE [41–45].

In this study, we also evaluated the effect of *S. aristata* extract in a murine model of CE. Administration of 50 mg/kg of *S. aristata* extract for 23 days in infected mice caused a significant decrease in the weight of the cysts compared with the control group and remarkable ultrastructural damage on the germinal layer. Similar results were obtained after treatment with 50 mg/kg of *S. multiaristata* extract for 20 days [14]. Our results are also consistent with those previously reported by other authors who used the methanolic extract of Zataria multiflora and the aqueous extract of *Sophora moorcroftiana* seeds [43, 54].

On the other hand, mice showed no undesirable side effects during the treatment period. We observed no differences between control and treated animals in ALP, GGT, and GPT enzyme activity, indicating no hepatotoxic effect.

Among the phytochemical groups that form the genus Stevia are the sesquiterpene lactones, diterpenoids, and flavonoids, among others [13]. In particular, the chemical composition of *S. aristata* was published by Zdero et al. [46] in which beyerene derivatives and other terpenoids were reported. *In vitro* antiparasitic efficacy has been described for many compounds belonging to these groups [13, 47–49]. Interestingly, the effect of the sesquiterpene lactone eupatoriopicrin was determined on an in vivo model of T. cruzi infection [50]. With regards to anti-Echinococcus activity, *Capparis spinosa* extract has shown *in vitro* and ex vivo protoscolicidal effects on *E. granulosus*. In this extract terpenoid and phenolic compounds have been detected [51]. On the other hand, the terpenoid crocin exerted *in vitro* and in vivo activity against *E. multilocularis* [52].

In conclusion, we demonstrated that the dichloromethane extracts of *S. satureiifolia, S. aristata, G*. *pulchella,* and *G. chiloensis* caused a reduction in protoscoleces viability. Moreover, *S. aristata* extract produced an *in vitro* effect on *E. granulosus* cysts and, also, a better pharmacotherapeutic efficacy than the reference drug, albendazole. These findings highlight the importance of *S. aristata* in the search for new antiechinococcal compounds. Likewise, due to the promising results reported with the use of *S. rebaudiana* on gastrointestinal parasites both in adults and children and the fact that no side effects were noted for this research, as in our study using the murine model, in the future, it could be considered a study of the antiparasitic efficacy of *S. aristata* on humans.

## Figures and Tables

**Table 1:** Clinical efficacy study. Median weight (*g*) and interquartile range (IQR) of *E. granulosus* cysts were determined in experimentally infected mice from the unmedicated and treated groups.

Group	Median weight of cysts (*g*)	Interquartile range (IQR)
Control	5.47	5.385
ABZ	3.75	1.705
*S. aristata*	2.55^*∗*^	3.51

^*∗*^Statistically significant differences with the control group (*P* < 0.05).

## Data Availability

The data are available upon request.

## Additional Points

(i) The *in vitro* efficacy of Asteraceae family plant extracts on protoscoleces was demonstrated. (ii) *S. aristata* extract showed the highest effect on protoscoleces compared with other extracts. (iii) *S. aristata* extract induced a rapid germinal layer collapse in metacestodes. (iv) The *in vivo* treatment with *S. aristata* extract caused a reduction in the weight of the cysts and damage to the germinal layer.

## References

[1] Romig T., Deplazes P., Jenkins D. (2017). Ecology and life cycle patterns of Echinococcus species. *Advances in Parasitology*.

[2] Tamarozzi F., Deplazes P., Casulli A. (2020). Reinventing the wheel of Echinococcus granulosus sensu lato transmission to humans. *Trends in Parasitology*.

[3] Budke C. M., Deplazes P., Torgerson P. R. (2006). Global socioeconomic impact of cystic echinococcosis. *Emerging Infectious Diseases*.

[4] Siles-Lucas M., Casulli A., Cirilli R., Carmena D. (2018). Progress in the pharmacological treatment of human cystic and alveolar echinococcosis: compounds and therapeutic targets. *PLoS Neglected Tropical Diseases*.

[5] Wen H., Vuitton L., Tuxun T. (2019). Echinococcosis: advances in the 21st century. *Clinical Microbiology Reviews*.

[6] Hemphill A., Stadelmann B., Scholl S. (2010). Echinococcus metacestodes as laboratory models for the screening of drugs against cestodes and trematodes. *Parasitology*.

[7] Vuitton D. A. (2009). Benzimidazoles for the treatment of cystic and alveolar echinococcosis: what is the consensus?. *Expert Review of Anti-infective Therapy*.

[8] Wang S., Ma Y., Wang W. (2022). Status and prospect of novel treatment options toward alveolar and cystic echinococcosis. *Acta Tropica*.

[9] Ali R., Khan S., Khan M. (2020). A systematic review of medicinal plants used against Echinococcus granulosus. *PLoS One*.

[10] Rolnik A., Olas B. (2021). The plants of the asteraceae family as agents in the protection of human health. *International Journal of Molecular Sciences*.

[11] Kinghorn D. (2002). Overview. *Stevia: The Genus Stevia*.

[12] Soejarto D. D., Kinghorn D. (2002). Botany of Stevia and Stevia rebaudiana. *Stevia: The Genus Stevia*.

[13] Borgo J., Laurella L. C., Martini F., Catalán C. A. N., Sülsen V. P. (2021). Stevia genus: phytochemistry and biological activities update. *Molecules*.

[14] Albani C. M., Borgo J., Fabbri J. (2021). Anthelmintic activity of Stevia multiaristata extract against Echinococcus granulosus sensu stricto. *Parasitology*.

[15] Fabbri J., Clemente C. M., Elissondo N. (2020). Anti-echinococcal activity of menthol and a novel prodrug, menthol-pentanol, against Echinococcus multilocularis. *Acta Tropica*.

[16] Elissondo M., Dopchiz M., Ceballos L. (2006). *In vitro* effects of flubendazole on Echinococcus granulosus protoscoleces. *Parasitology Research*.

[17] Casado N., Rodríguez-Caabeiro F., Hernández S. (1986). *In vitro* survival of Echinococcus granulosus protoscolices in several media, at +4 degrees C and +37 degrees C. *Zeitschrift für Parasitenkunde*.

[18] Cucher M., Prada L., Mourglia-Ettlin G. (2011). Identification of Echinococcus granulosus microRNAs and their expression in different life cycle stages and parasite genotypes. *International Journal for Parasitology*.

[19] Elissondo M., Ceballos L., Dopchiz M. (2007). *In vitro* and *in vivo* effects of flubendazole on Echinococcus granulosus metacestodes. *Parasitology Research*.

[20] Bessey O. A., Lowry O. H., Brock M. J. (1946). A method for the rapid determination of alkaline phosphatase with five cubic millimeters of serum. *Journal of Biological Chemistry*.

[21] Bergmeyer H. U., Bowes G., Horder M., Moss D. W. (1976). Provisional recommendations on IFCC methods for the measurement of catalytic concentrations of enzymes. Part 2. IFCC method for aspartate aminotransferase. *Clinica chimica acta; international journal of clinical chemistry*.

[22] Shaw L. M., Stromme J. H., London J. L., Theodorsen L. (1983). International Federation of Clinical Chemistry, (IFCC), Scientific Committee, Analytical Section. IFCC methods for the measurement of catalytic concentration of enzymes. Part 4. IFCC method for gamma-glutamyltransferase [(gamma-glutamyl)-peptide: amino acid gamma-glutamyltransferase, EC 2.3.2.2]. *Journal of clinical chemistry and clinical biochemistry. zeitschrift fur klinische chemie und klinische biochemie*.

[23] Szasz G. (1969). A kinetic photometric method for sérum ɤ-glutamyl transpeptidase. *Clinical Chemistry*.

[24] Fox J., Weisberg S. (2019). *An {R} Companion to Applied Regression*.

[25] Lenth R. V. (2021). Emmeans: estimated marginal means, aka least-squares means. https://CRAN.R-project.org/package=emmeans.

[26] Alves K. S. (2020). ec50estimator: An automated way to estimate EC50 for stratified datasets. https://CRAN.R-project.org/package=ec50estimator.

[27] R Core Team (2021). *R: A Language and Environment for Statistical Computing*.

[28] Craig P. S., Budke C. M., Schantz P. M. (2007). Human echinococcosis: a neglected disease?. *Tropical Medicine and Health*.

[29] Cos P., Vlietinck A. J., Berghe D. V., Maes L. (2006). Anti-infective potential of natural products: how to develop a stronger *in vitro* “proof-of-concept”. *Journal of Ethnopharmacology*.

[30] Bohm B., Stuessy T. (2001). *Flavonoids of the Sunflower Family (Asteraceae)*.

[31] Koc S., Isgor B. S., Isgor Y. G., Shomali Moghaddam N., Yildirim O. (2015). The potential medicinal value of plants from Asteraceae family with antioxidant defense enzymes as biological targets. *Pharmaceutical Biology*.

[32] Ruiz-Ruiz J. C., Moguel-Ordoñez Y. B., Segura-Campos M. R. (2017). Biological activity of stevia rebaudiana bertoni and their relationship to health. *Critical Reviews in Food Science and Nutrition*.

[33] Achucarro C., Ferro E. A., Richer Y. (2011). Preliminary clinical evaluation of the antiparasite effect of Stevia rebaudiana Bertoni (ka`a he ê) in adults and children. *Anales de la Facultad de Ciencias Médicas*.

[34] Elissondo M. C., Pensel P. E., Denegri G. M. (2013). Could thymol have effectiveness on scolices and germinal layer of hydatid cysts?. *Acta Tropica*.

[35] Fabbri J., Maggiore M. A., Pensel P. E., Denegri G. M., Gende L. B., Elissondo M. C. (2016). *In vitro* and *in vivo* efficacy of carvacrol against echinococcus granulosus. *Acta Tropica*.

[36] Pensel P. E., Maggiore M. A., Gende L. B., Eguaras M. J., Denegri M. G., Elissondo M. C. (2014). Efficacy of essential oils of thymus vulgaris and origanum vulgare on echinococcus granulosus. *Interdisciplinary Perspectives on Infectious Diseases*.

[37] Amiri K., Nasibi S., Mehrabani M., Nematollahi M. H., Harandi M. F. (2019). *In vitro* evaluation on the scolicidal effect of myrtus communis L. and Tripleurospermum disciforme L. methanolic extracts. *Experimental Parasitology*.

[38] Bouaziz S., Amri M., Taibi N. (2021). Protoscolicidal activity of Atriplex halimus leaves extract against Echinococcus granulosus protoscoleces. *Experimental Parasitology*.

[39] Cheraghipour K., Beiranvand M., Zivdari M. (2021). *In vitro* potential effect of Pipper longum methanolic extract against protoscolices of hydatid cysts. *Experimental Parasitology*.

[40] Tabatabaei Z. S., Dehshahri S., Taghi M. M. (2019). *In vitro* study on protoscolicidal effect of methanolic extract of allium hirtifolium on protoscoleces of cystic echinococcosis. *Infectious Disorders - Drug Targets*.

[41] Haji Mohammadi K. H., Heidarpour M., Borji H. (2018). *In vivo* therapeutic efficacy of the allium sativum ME in experimentally echinococcus granulosus infected mice. *Comparative Immunology, Microbiology and Infectious Diseases*.

[42] Labsi M., Khelifi L., Mezioug D., Soufli I., Touil-Boukoffa C. (2016). Antihydatic and immunomodulatory effects of Punica granatum peel aqueous extract in a murine model of echinococcosis. *Asian Pacific Journal of Tropical Medicine*.

[43] Luo Y., Zhang G., Liu X. (2018). Therapeutic and immunoregulatory effects of water-soluble alkaloids E2-a from sophora moorcroftiana seeds as a novel potential agent against echinococcosis in experimentally protoscolex-infected mice. *Veterinary Research*.

[44] Lv H., Jiang Y., Liao M., Sun H., Zhang S., Peng X. (2013). *In vitro* and *in vivo* treatments of echinococcus granulosus with huaier aqueous extract and albendazole liposome. *Parasitology Research*.

[45] Moazeni M., Larki S., Saharkhiz M. J., Oryan A., Ansary Lari M., Mootabi Alavi A. (2014). *In vivo* study of the efficacy of the aromatic water of zataria multiflora on hydatid cysts. *Antimicrobial Agents and Chemotherapy*.

[46] Zdero C., Bohlmann F., Schmeda-Hirschmann G. (1987). Beyerene derivatives and other terpenoids from stevia aristata. *Phytochemistry*.

[47] Beer M. F., Frank F. M., Germán Elso O. (2016). Trypanocidal and leishmanicidal activities of flavonoids isolated from Stevia satureiifolia var. satureiifolia. *Pharmaceutical Biology*.

[48] Sülsen V. P., Lizarraga E. F., Elso O. G. (2019). Activity of estafietin and analogues on trypanosoma cruzi and leishmania braziliensis. *Molecules*.

[49] Sülsen V. P. (2021). Sesquiterpene lactones and diterpenes: promising therapeutic candidates for infectious diseases, neoplasms and other chronic disorders. *Molecules*.

[50] Elso O. G., Bivona A. E., Sanchez Alberti A. (2020). Trypanocidal activity of four sesquiterpene lactones isolated from asteraceae species. *Molecules*.

[51] Mahmoudvand H., Khalaf A. K., Beyranvand M. (2021). *In vitro* and *ex vivo* evaluation of Capparis spinosa extract to inactivate protoscoleces during hydatid cyst surgery. *Current Drug Discovery Technologies*.

[52] Liu C., Fan H., Guan L., Ge R. L., Ma L. (2021). *In vivo* and *in vitro* efficacy of crocin against echinococcus multilocularis. *Parasites & Vectors*.

[53] Fabbri J., Maggiore M. A., Pensel P. E., Denegri G. M., Elissondo M. C. (2020). *In vitro* efficacy study of Cinnamomum zeylanicum essential oil and cinnamaldehyde against the larval stage of echinococcus granulosus. *Experimental Parasitology*.

[54] Moazeni M., Larki S., Oryan A., Saharkhiz M. J. (2014). Preventive and therapeutic effects of zataria multiflora methanolic extract on hydatid cyst: an *in vivo* study. *Veterinary Parasitology*.

[55] Zuloaga F., Morrone O., Belgrano M. (2008). Catálogo de las Plantas Vasculares del Cono Sur (Argentina, Sur de Brasil, Chile, Paraguay y Uruguay). *Dicotyledoneae: Acanthaceae-Fabaceae (Abarema-Schizolobium)*.

